# Enhanced recognition of human colorectal tumour cells using combinations of monoclonal antibodies.

**DOI:** 10.1038/bjc.1989.379

**Published:** 1989-12

**Authors:** L. G. Durrant, R. A. Robins, K. C. Ballantyne, R. A. Marksman, J. D. Hardcastle, R. W. Baldwin

**Affiliations:** Cancer Research Campaign Laboratory, University of Nottingham, UK.

## Abstract

Murine monoclonal antibodies directed against tumour associated antigens are potentially useful in tumour diagnosis and therapy. However, all the antigens they recognise may be heterogeneously expressed on tumours and this may allow escape of cells from therapy if a single monoclonal antibody is used. One approach is to use combinations of monoclonal antibodies recognising complementary cell surface antigens. A flow cytometric method which allows accurate quantitation of the intensity of staining and the percentage of fresh primary tumour cells binding a series of monoclonal antibodies has therefore been developed. This allows calculations as the number of drug molecules which could be potentially delivered by each monoclonal antibody and the optimal combination of antibodies which should be used. Monoclonal antibodies recognising Y hapten (C14), CEA (228, 161) and 791T-p72 antigen (791T/36) have been screened as a possible combination for colorectal cancer. There was inter-tumour variation in the binding of all the monoclonal antibodies although combinations could reduce or abrogate this problem. A combination of the monoclonal antibodies C14, 228, 791T/36 and 161 would recognise 100% of tumours. Sixty per cent of tumours bound all four antibodies, 78% any three, 90% any two and 100% any one antibody. There was also intra-tumour variation in the number of tumour cells per lesion that were recognised, the best monoclonal antibody, 161, stained a mean of 59% of cells per tumour whereas the anti-cytokeratin monoclonal antibody stained a mean of 74% of cells per tumour. An increased intensity of staining of tumour membranes was observed when a combination of C14 and 228 was used compared to binding of individual antibodies. Furthermore there was still no significant binding to normal colon membranes. Combinations of monoclonal antibodies which recognise a high percentage of tumours are likely to be necessary for monoclonal antibody drug targeting to prevent tumour recurrence and/or metastases.


Br. J. Cancer (1989), 60, 855-860 © The Macmillan Press Ltd., 1989

### Enhanced recognition of human colorectal tumour cells using combinations of monoclonal antibodies

L.G. Durrant[1], R.A. Robins[1], K.C. Ballantyne[2], R.A. Marksman[1], J.D. Hardcastle[2] & R.W. Baldwin[1]

[1]*Cancer Research Campaign Laboratories, University of Nottingham, Nottingham NG7 2RD, UK; and* [2]*Department of Surgery, University Hospital, Nottingham NG7 2UH, UK.*

**Summary** Murine monoclonal antibodies directed against tumour associated antigens are potentially useful in tumour diagnosis and therapy. However, all the antigens they recognise may be heterogeneously expressed on tumours and this may allow escape of cells from therapy if a single monoclonal antibody is used. One approach is to use combinations of monoclonal antibodies recognising complementary cell surface antigens. A flow cytometric method which allows accurate quantitation of the intensity of staining and the percentage of fresh primary tumour cells binding a series of monoclonal antibodies has therefore been developed. This allows calculations as the number of drug molecules which could be potentially delivered by each monoclonal antibody and the optimal combination of antibodies which should be used. Monoclonal antibodies recognising Y hapten (C14), CEA (228, 161) and 791T-p72 antigen (791T/36) have been screened as a possible combination for colorectal cancer. There was inter-tumour variation in the binding of all the monoclonal antibodies although combinations could reduce or abrogate this problem. A combination of the monoclonal antibodies C14, 228, 791T/36 and 161 would recognise 100% of tumours. Sixty per cent of tumours bound all four antibodies, 78% any three, 90% any two and 100% any one antibody. There was also intra-tumour variation in the number of tumour cells per lesion that were recognised, the best monoclonal antibody, 161, stained a mean of 59% of cells per tumour whereas the anti-cytokeratin monoclonal antibody stained a mean of 74% of cells per tumour. An increased intensity of staining of tumour membranes was observed when a combination of C14 and 228 was used compared to binding of individual antibodies. Furthermore there was still no significant binding to normal colon membranes. Combinations of monoclonal antibodies which recognise a high percentage of tumours are likely to be necessary for monoclonal antibody drug targeting to prevent tumour recurrence and/or metastases.

A variety of monoclonal antibodies reactive with colorectal tumour associated antigens have been described (Steplewski *et al.*, 1982; Herlyn *et al.*, 1979; Lindholm *et al.*, 1983; Durrant *et al.*, 1986*a*). However, one problem that needs to be solved before monoclonal antibodies can be used effectively for imaging or therapy is the heterogeneity of cell surface antigen expression on tumours (Durrant *et al.*, 1986*a*; Brattain *et al.*, 1981; Dexter *et al.*, 1981). Variation in antigen expression and density on individual tumour cells from primary and metastatic tumours (Ballantyne *et al.*, 1986) could account in part for the difficulties in detecting all known lesions *in vivo* using a single radiolabelled monoclonal antibody and could lead to escape from the cytotoxic effects of a single monoclonal antibody-drug conjugate. One approach to these problems is to use a combination of mono clonal antibodies which recognise distinct surface antigens and/or epitopes covering the range of heterogeneous antigen expression.

Although this is not a new concept very few studies have been published showing that combinations of antibodies recognise a larger number of primary tumours. Most studies have used either cell lines or human xenografts, neither of which has the heterogeneity observed in human tumours. Our early studies (Durrant *et al.*, 1986*a*) showed that it was necessary to analyse at least 50 individual colorectal tumours to obtain a clear picture of tumour phenotypes as the inter-tumour variation was so diverse. In this report we compared the binding of a panel of anti-colorectal monoclonal antibodies reactive with different 'tumour associated' epitopes singly and in combinations, to colorectal tumour cells.

The monoclonal antibodies have been selected because they bind preferentially to tumour cells compared to a panel of normal tissues as assayed by immunocytochemical staining of cryopreserved tissues. 791T/36 recognises a glycoprotein of molecular weight 72,000 which is expressed on colorectal osteosarcoma and ovarian tumours. Its main reactivity with normal tissues is with activated T lymphocytes and stromal tissues (Embleton *et al.*, 1981; Price *et al.*, 1983*a*, *b*). It has been used extensively for colorectal tumour imaging (Farrands *et al.*, 1982; Armitage *et al.*, 1983) and linked to ricin A chain has been screened in a phase 1 clinical trial (Byers *et al.*, 1989). 228 recognises carcinoembryonic antigen and not normal cross-reacting antigen. Its main normal reactivity is with secretory components of the gastrointestinal tract. It has also been shown to localise in tumours of colorectal cancer patients (Pimm *et al.*, 1986). 161 antibody also recognises CEA but also cross-reacts with NCA. It therefore binds to a number of normal tissues, including liver and kidney tissues and would therefore be of limited clinical value. C14 recognises the Y haptenic blood group antigen which is expressed widely on colorectal cancers and adenomas (Lloyd *et al.*, 1983; Brown *et al.*, 1984; Abe *et al.*, 1986; Durrant *et al.*, 1986*a*, *b*; Ernst *et al.*, 1986). Its main reactivity with normal tissues is restricted to secretory epithelial tissues in individuals who secrete blood group substances (Brown *et al.*, 1984).

Although immunoperoxidase staining of tumour cells by monoclonal antibodies was ideal for showing spatial relationships between cells it could not quantitate cell surface expression and was extremely time consuming for calculating the number of positive cells. A quantitative flow cytometric technique for rapid analysis of cell surface antigen expression on cell suspensions from solid tumours has therefore been developed. The relative binding of combinations of antibodies as compared to single antibodies has also been quantitatively compared by ELISA on tumour and normal extranuclear membrane extracts.

Our results indicate that more colorectal cancers were recognised and the intensity of staining was increased by using combinations of monoclonal antibodies when compared to a single antibody.

### Materials and methods

### *Tumour cells*

Cell suspensions were prepared from tissue of 50 individual tumours within 18 h of removal. Tissue was finely minced

Correspondence: L.G. Durrant.
Received 27 January 1989; and in revised form 6 June 1989.

and disaggregated in 0.05% collagenase (Boehringer, Mannheim, FR Germany) as previously described (Durrant et al., 1986a). This collagenase had no effect on antigen expression on cultured cell lines. The clinicopathology of these tumours is shown in Table I.

*Monoclonal antibodies*

*Antibodies to tumour associated antigens* A panel of murine monoclonal antibodies were used in this study. 791T/36 is an $IgG_2b$ monoclonal antibody which recognises the 791T p72 antigen found in many carcinomas (Embleton et al., 1981; Price et al., 1983). C14 is an IgM monoclonal antibody which recognises the Y haptenic blood group antigen expressed by the majority of colon adenocarcinomas (Brown et al., 1983). 161 is an $IgG_1$ monoclonal antibody that recognises a similar epitope expressed on CEA and NCA (Price et al., 1987). Finally, 228 monoclonal antibody recognised an epitope only expressed on CEA (Price et al., 1987).

*Antibodies to normal tissue components* F15-42 reactive with human Thy 1 antigen (McKenzie & Fabre, 1981) and F10-89-4 (Dalchau et al., 1980) reactive with human leukocyte common antigen were obtained from Serotec Ltd (Bicester, UK). These antibodies recognise stromal cells and leukocytes respectively. Monoclonal antibody Cam 5.2 (Becton Dickinson, Oxnard, CA, USA) recognises cytokeratin, a cytoskeletal component of all epithelial cells (Makin et al., 1984).

*Indirect immunofluorescence*

Cells were washed twice in Hank's balanced salt solution (HBSS) and stained by indirect immunofluorescence as previously described (Durrant et al., 1986a, b). All the monoclonal antibodies, singly and in combinations, were used at $100\ \mu g\ ml^{-1}$, $10\ \mu g$ per tube which has been shown to be an excess of antibody by titration experiments on cells expressing each antigen at high density. Cells staining with Cam 5.2, which recognises an internal antigen, were pre-fixed with paraformaldehyde (1%, 20 min). The analysis was restricted to the size range of malignant cells by appropriate forward angle scatter gating. Fluorescence intensity is expressed as a mean channel number (mean linear fluorescence, MLF) calculated by multiplying the contents of each channel by its channel number and dividing by the total number of cells in the distribution. Each tumour and cell line was also stained using normal mouse immunoglobulin and the MLF in this control was subtracted from the values obtained with monoclonal antibody. However, the MLF of binding of normal mouse immunoglobulin was $58 \pm 32$ and therefore tumours were only described as staining significantly if the MLF exceeds 58 + two standard deviations, i.e. 122.3. This was a conservative estimated as background levels with normal mouse immunoglobulin have already been subtracted. The percentage of positively stained cells was calculated as the number of cells with a fluorescence that exceeded the value in which 95% of cells staining with normal mouse immunoglobulin were observed.

**Table I** Clinicopathology of colorectal tumours

| *Stage*[a] | | *Grade*[b] | | *Site* | | *Ploidy*[c] | |
|---|---|---|---|---|---|---|---|
| *Disaggregated tumours* | | | | | | | |
| A | 26% | Vad. | 4% | Colon | 42% | A | 52% |
| B | 30% | W | 14% | Rectum | 58% | D | 48% |
| C | 20% | M | 64% | | | | |
| D | 24% | P | 18% | | | | |
| *Tumour ENM preparations* | | | | | | | |
| A | 7% | Vad. | 0% | Colon | 58% | A | 42% |
| B | 25% | W | 0% | Rectum | 42% | D | 58% |
| C | 25% | M | 75% | | | | |
| D | 33% | P | 25% | | | | |

[a]Dukes' staging with stage D describing tumours with distant metastases. [b]Vad., villous adenoma. The high percentage of A tumours is due to the active screening programme in Nottingham. W, well differentiated; M, moderately differentiated; P, poorly differentiated. [c]Ploidy A, aneuploid tumours were stained with propidium iodide and analysed flow cytometrically. Tumours were aneuploid if their DNA index was between 1.1 and 1.9 and greater than 10% of the total cells produced the abnormal G0/G1 peak or if the index was between 1.9 and 2.1 and greater than 15% of the total cells produced the second peak. D, diploid.

*Extranuclear membrane preparations*

Fresh surgically resected colorectal adenocarcinomas or normal colonic mucosa were homogenised in a buffer containing 1 mM $NaHCO_3$, 2 mM $CaCl_2$, 2 mM $MgCl_2$, 1 mM phenylmethylsulphonyl fluoride pH 7.6 (4 vol. of buffer per g of tissue) to prepare a crude membrane preparation as described previously (Brown et al., 1983). Membranes were prepared from 12 individual tumours. The clinicopathology of these tumours is shown in Table I.

*Solid phase enzyme linked immunoadsorbent assay*

Extranuclear membranes were diluted to $0.2\ mg\ ml^{-1}$ protein in phosphate buffered saline (PBS) and $25\ \mu l$ aliquots were added per well in type 3912 polyvinylchloride (PV) microtitre plates (Becton Dickinson, CA, USA). After an 18 h incubation at 4°C the wells were washed in PBS containing 0.05% Tween 20 (washing buffer) and filled with PBS containing 1% BSA. After 1 h at 4°C the wells were emptied and washed twice with washing buffer followed by addition of $25\ \mu l$ of hybridoma supernatant per well. After 1 h at room temperature the wells were washed five times with washing buffer and then $150\ \mu l$ of goat IgG anti-mouse Ig (Sigma, Poole, UK) conjugated to alkaline phosphatase was added for a further 1 h. Wells were washed five times in washing buffer and $50\ \mu l$ of Sigma 104 phosphatase substrate (Sigma, Poole), diluted to $1\ mg\ ml^{-1}$ in substrate buffer, was added. The absorbence of each well was read after 45 min at 405 nm in a Titertek multiscan.

### Results

*Disaggregation of solid tumours*

Dissaggregation of solid tumours yields a mixed population of cells including red blood cells, lymphocytes, stromal cells, macrophages and endothelial cells. The percentage of epithelial cells, as measured by staining of cytokeratin with monoclonal antibody Cam 5.2, was only $22 \pm 13\%$ (range 10-80). However, following forward angle light scatter gating to selectively analyse cells in the malignant cell size range $79 \pm 4\%$ (range 69-86) of the cells analysed were epithelial. Furthermore the variation between tumours was considerably reduced.

The percentage of lymphocytes, as measured by staining with the monoclonal antibody F10-89-4, in the total nucleated population was $74 \pm 16\%$ (range 40-90). This was considerably reduced to $5.5 \pm 5\%$ (range 1-20) following FACS IV gating for malignant cell size. The percentage of stromal cells in the population of cells analysed in the malignant size range was $3.5 \pm 3\%$ (range 1-13).

The percentage of non-epithelial cells in the forward light scatter gate was low, and it did not vary considerably between tumours ($21 \pm 4\%$). If the monoclonal antibodies cross-reacted with these cells it may have contributed to the intensity of staining. However, only monoclonal antibody 791T/36 stained any of the normal cells found within the tumours. This antibody stains stromal cells but no distinct peak of fluorescence could be detected on careful analysis of the fluorescence profiles, suggesting that these cells do not affect the mean intensity of staining.

### *Antigen expression on tumour cells*

Expression of CEA and the Y hapten on colorectal tumours have previously been shown by immunoperoxidase staining of cryopreserved tissue. However, there are advantages in analysing tumour cell suspensions by indirect immunofluorescence and flow cytometric analysis. A large proportion of the tumour (1–5 g of tissue was disaggregated, a minimum of $10^4$ epithelial cells were processed per antibody) was analysed, which allows accurate quantification of the mean antibody binding level per cell for each individual tumour and the percentage of cells within a tumour which bind each monoclonal antibody.

Analysis of the binding of a panel of monoclonal antibodies, recognising tumour associated antigens, to a series of disaggregated colorectal tumours illustrates the inter-tumour variation in intensity of staining (Figure 1) and the intra-tumour variation in the percentage of cells expressing the relevant antigens (Figure 2).

All of the monoclonal antibodies recognising tumour associated antigens stained the majority of tumours with a moderate intensity (MLFs of 122–1,000). However, monoclonal antibody 161 stained the most tumours (36%) with strong intensity (MLF > 1,000), monoclonal antibodies C14 and 228 both stained 26% of tumours strongly whereas 791T/36 failed to stain any tumours strongly. The monoclonal antibodies recognising stromal cells and the lymphocytes both stained cells in the malignant cell size range weakly (MLF > 122). In contrast the monoclonal antibody, Cam5.2, recognising epithelial cells bound 58% of the tumours moderately and 42% strongly.

None of the monoclonal antibodies stained all the tumour cells within every lesion. Indeed the best, 161, stained a mean of 59% of cells within a tumour, whereas the monoclonal antibody Cam5.2, which recognised epithelial cells, stained a mean of 74% of the cells per tumour. Furthermore, none of the monoclonal antibodies stained cells within all of the tumours, 791T/36 stained cells within 64% of the tumours, C14 stained 83%, 228 stained 82% and 161 stained 90%. Any single monoclonal antibody therefore failed to react with between 10 and 36% of the colorectal tumours.

**Figure 1** Binding of monoclonal antibodies to freshly disaggregated colorectal tumour cells as stained by indirect immunofluorescence and analysed by flow cytometry. Each point refers to an individual tumour.

**Figure 2** Percentage of cells within a tumour recognised by a series of monoclonal antibodies. Disaggregated colorectal tumours are stained by indirect immunofluorescence and analysed by flow cytometry. Each point refers to an individual tumour.

### *Immunophenotypes*

Immunophenotypes of colorectal tumours were analysed to determine if an appropriate combination of monoclonal antibodies could stain all of the tumours (Figure 3). Cell suspensions from each tumour were stained separately with each of the monoclonal antibodies. If a population stained significantly with a monoclonal antibody (see Materials and methods) then the tumour was assigned to the relevant immunophenotype. If a tumour only stained with C14 then it

**Figure 3** Colorectal tumour immunophenotypes. Cell suspensions from each tumour were stained separately with each of the monoclonal antibodies. If a population stained significantly with a monoclonal antibody (see Materials and methods) then the tumour was assigned to the relevant immunophenotype. If a tumour only stained with C14 then that is its phenotype. However, if some cells stained with C14 and/or 161 then it would have a C14, 161 phenotype. The figures inside the circles refer to the percentage of 50 tumours in which some of their cells stained with the designated antibodies.

was designated as a C14 phenotype (i.e. 6% of the tumours). However, if some of the tumour cells stained with C14 and/or 161 then it would have a C14, 161 phenotype (i.e. 6% of the tumours). Ten per cent of the tumours expressed only a single epitope and therefore the monoclonal antibodies reacting with these epitopes must be included in any combination if all tumours are to be recognised. Table II computes the potential effectiveness of various optimum combinations of all four monoclonal antibodies, 161, 228, C14 and 791T/36, in a series of 50 colorectal tumours. The best combination would consist of all four monoclonal antibodies as this would recognise all of the colorectal tumours, 60% binding all four, 18% any three, 12% any two and 10% binding a single monoclonal antibody. However, a combination excluding antibody 161 which cross-reacts with NCA found on normal granulocytes would still be effective against 98% of the tumours, 60% reacting with all three monoclonal antibodies and 80% with any two. A combination of C14 and 228 would also stain 98% of the tumours; 70% of these would react with both monoclonal antibodies.

*Binding to extranuclear membranes*

Binding of a panel of four monoclonal antibodies, 791T/36, C14, 228 and 161, to a panel of 12 tumour and autologous normal membrane preparations were assessed. Significant binding to normal colon membranes was only observed for monoclonal antibody 161 and it has therefore been excluded from these studies. Results of the binding of the other three monoclonal antibodies singly and in combinations are shown in Table III.

C14 monoclonal antibody bound with the strongest intensity when compared to 791T/36 ($P<0.01$) or to 288 ($P<0.05$). However, a combination of C14 and 228 stained significantly stronger than either C14 ($P<0.01$) or 228 ($P<0.01$). Furthermore the combination stained all of the membranes. The addition of 791T/36 failed to significantly increase the intensity of staining seen with C14 and/or 228. Of particular interest was the tumour (T8) which only stained weakly with the monoclonal antibody C14 and failed to stain significantly above background with the monoclonal antibodies 228 and 791T/36. All the combinations bound significantly but a combination of 228 and C14 bound 2.2 times more strongly than C14 alone. None of the combinations significantly stained autologous normal membranes.

**Table II** Optimum combinations of monoclonal antibodies which react with colorectal tumours

| *Monoclonal antibodies* | *% of tumours binding any one antibody* |
|---|---|
| C14 | 88 |
| 791T/36 | 70 |
| 228 | 80 |
| 161 | 90 |
| C14 and 228 | 98 (70)[a] |
| C14 and 791T/36 | 92 (66)[a] |
| 791T/36 and 228 | 86 (64)[a] |
| 791T/36, 228 and C14 | 98 (80)[a] (60)[b] |
| 791T/36, 228, C14 and 791T/36 | 100(90)[a] (78)[b] (60)[c] |

[a]% of tumours which bind two antibodies. [b]% of tumours which bind three antibodies. [c]% of tumours which bind four antibodies.

### Discussion

Murine monoclonal antibodies directed against tumour associated antigens are potentially useful for both tumour diagnosis and therapy. However, previous studies have shown qualitative differences in tumour associated antigen expression between tumours (Durrant *et al.*, 1986; Ballantyne *et al.*, 1986; Spremulli *et al.*, 1983) and within a tumour (Durrant *et al.*, 1986*b*; Petricciani, 1982). Furthermore, the level of antigen expression on individual cells within a tumour varies enormously (Durrant *et al.*, 1986*b*). This may lead to failure to detect all tumour lesions and/or escape of tumour cells within a tumour during therapy with a single monoclonal antibody. The purpose of this study was to establish if monoclonal antibodies, recognising colorectal tumour associated antigens, could reduce inter and intra-tumour heterogeneity when used in combinations rather than individually. A qualitative flow cytometric assay which rapidly processes large numbers of tumour cells ($10^4$ epithelial cells) has been developed (Durrant *et al.*, 1986*a*) and was chosen as the most suitable analysis for this study.

Inter-tumour heterogeneity was completely abrogated by using a combination of the monoclonal antibodies, 791T/36, 228, C14 and 161, as all 50 tumours studied were stained by one or more of these monoclonal antibodies. This combination excluding the monoclonal antibody 161 which recognises CEA but cross-reacts with NCA and therefore stains normal colon and granulocytes, recognised 49 of the 50 tumours analysed. Ninety-eight per cent of these lesions would therefore be recognised by this simple combination of three antibodies. Similarly, although both 228 and C14 recognised 92% of the tumour extranuclear membranes, a combination of both antibodies bound significantly to 100% of the membranes.

Monoclonal antibodies 791T/36 and 228 have been used for diagnostic imaging of colorectal cancer (Armitage *et al.*, 1983; Pimm *et al.*, 1986). As C14 is an IgM antibody its molecular size precludes efficient localisation in tumour. However, these studies clearly show that the Y hapten is an extremely important colorectal tumour associated antigen and that an IgG variant of C14 may be clinically valuable. To reduce non-specific toxicity the optimal drug delivery system requires cleavage of the drug after the conjugate has bound to the tumour associated antigen and internalised. The majority of large molecules are transported by constitutive pinocytosis (Besterman & Low, 1983). Both 791T/36 and 228 monoclonal antibodies enter tumour cells by this route (Garnett *et al.*, 1986*b*; Byers *et al.*, 1988). There is no reason to assume C14 antibody will not be internalised by a similar mechanism. The antigen density per cell is very important as it will determine the number of molecules of conjugate internalised. An MLF of 1,000 approximately corresponds to

**Table III** Binding of monoclonal antibodies singly or in combinations to the extranuclear membrane preparations of colorectal tumours

| | *Binding to extranuclear membranes as measured by ELISA (OD × 10)* | | | | | | | | | | | | *Non-parametric, paired Wilcoxon rank test* | | |
|---|---|---|---|---|---|---|---|---|---|---|---|---|---|---|---|
| *Monoclonal Antibody* | *T* | *T* | *T* | *T* | *T* | *T* | *T* | *T* | *T* | *T* | *T* | *T* | *C14* | *791T/36* | *228* |
| C14 | 697 | 1304 | 668 | 1000 | 460 | 781 | 286 | 140 | 274 | 1269 | 191 | 1211 | n.a. | $P<0.01$ | $P<0.05$ |
| 791T/36 | 114 | 239 | 251 | 290 | 280 | 398 | 152 | 21 | 46 | 114 | 223 | 246 | $P<0.01$ | n.a. | $P<0.01$ |
| 228 | 186 | 901 | 561 | 1008 | 420 | 644 | 490 | 108 | 175 | 327 | 2021 | 646 | $P<0.05$ | $P<0.01$ | n.a. |
| C14 + 791T/36 | 616 | 974 | 698 | 1007 | 590 | 873 | 314 | 254 | 46 | 1050 | 229 | 1197 | n.s. | $P<0.01$ | n.a. |
| 791T/36 + 228 | 244 | 1019 | 576 | 1077 | 470 | 649 | 417 | 205 | **286** | 244 | 2084 | 647 | n.a. | $P<0.01$ | n.s. |
| 228 + C14 | 634 | **1584** | **923** | **1273** | **610** | 1046 | **611** | **313** | 253 | **1460** | **2269** | 1431 | $P<0.01$ | n.a. | $P<0.01$ |
| 791T/36 + C14 + 228 | **732** | 1554 | 910 | **1273** | 520 | **1103** | 544 | 279 | **286** | 1158 | 2152 | **1536** | $P<0.01$ | $P<0.01$ | $P<0.01$ |
| nMse Ig | 104 | 241 | 262 | 287 | 260 | 126 | 80 | 104 | 103 | 114 | 181 | 108 | $P<0.01$ | $P<0.01$ | $P<0.01$ |

Mean optical density of quadruplicate wells was read at 405nm. The standard error of the mean was less than 5% for all these results. Normal mouse immunoglobulin was included as a negative control. n.a., not applicable; n.s., not significant. The strongest binding for each tumour is shown in bold.

50,000 molecules of antibody bound per cell assuming that an average of two molecules of FITC conjugated mouse Ig binds to each monoclonal antibody molecule. The fluorescence to protein ratio of the anti-mouse sera was 2.3 and under the analysis conditions used there are 2,200 FITC molecules per channel. Any monoclonal antibody substituted with two molecules of drug which bound with an MLF of 1,000 would only have to internalise antigen conjugate complex once in order to internalise one million molecules of drug. This is the number of molecules of methotrexate that had to be internalised to kill 50% of the colorectal cancer cells in an *in vitro* cytotoxicity assay (Durrant & Garnett, unpublished results). Monoclonal antibody 161 stained 36% of the tumours with this intensity whereas monoclonal antibodies 228 and C14 both stained 26%. If a higher percentage of tumours are to be efficiently killed by drugs such as methotrexate, both repeated exposure and continuous internalisations would be necessary. Alternatively, drugs could be attached to combinations of antibodies which increase the intensity of binding, more cytotoxic agents such as ricin toxin (Casellas *et al.*, 1984) could be conjugated to antibody or more molecules of a drug could be attached via a carrier to each antibody molecule (Garnett & Baldwin, 1986). The disadvantage of the latter two methods is that if the tumour associated antigen is only expressed weakly on normal cells sufficient drug will accumulate to cause cytotoxicity.

The intensity of staining tumour membranes was increased without significant binding to autologous normal colon membranes using combinations of either 228 and C14 or of 228, C14 and 791T/36 when compared to binding of each individual monoclonal antibody. In fact, one tumour which failed to bind significantly to any single monoclonal antibody stained significantly with a combination of 228 and C14. Similar *in vitro* studies in our laboratory have shown that two high affinity anti-CEA antibodies, 198 and B14/B8, potentiated the binding of 228 monoclonal antibody to the surface of MKN45 cells by increasing the dwell time of the cell bound antibody. This in turn led to increased endocytosis of the antibody and when 228 was linked to ricin A chain produced enhanced cytotoxicity (Byers *et al.*, 1988). As the spectrum of antigen expression is different on normal tissues these effects may not be seen on healthy tissue and therefore combinations of monoclonal antibodies may increase the tumour to normal binding ratios. The only normal tissue which more than one of the antibodies cross-react with by immunohistology is gastrointestinal epithelium. No significant binding to normal colon membranes of any of the monoclonal antibodies, either alone or in combinations, was detected, probably because CEA and the antigen bearing the Y hapten are exclusively expressed as secretory products and not membrane antigens in normal tissue. Viable epithelial cells from normal colon are difficult to prepare, but limited flow cytometric tests with cells derived from non-tumour tissue in resected colon tumour specimens confirm this low level of reactivity. Thus 791T/36 was unreactive in 3/3 tests; anti-CEA antibodies gave a MLF of 18.4 (five tests) and C14 an MLF of 71.9 (three tests) above a background MLF of 48.1.

Further studies using FITC labelled monoclonal antibodies to tumour associated antigens, and biotin labelled anti-cytokeratin antibodies and avidin phycoerythrin to identify epithelial cells will allow direct measurements of any increase in the intensity of staining and the percentage of cells recognised using combinations of antibodies. Krizan *et al.* (1985) showed that both the relative fluorescence and the percentage of cells recognised was increased when suboptimal doses of three monoclonal antibodies were added to cells rather than saturating concentrations of each monoclonal antibody alone or combined. Perhaps the total dose of monoclonal antibody can be reduced by using combinations.

Ceriani *et al.* (1987) demonstrated that a combination of antibodies recognising different epitopes on the same antigen could increase the therapeutic effect against a relevant human xenograft, but not against an antigen negative xenograft, when compared to single antibody studies.

If combinations of monoclonal antibodies were used clinically these studies predict that all lesions could be recognised and the intensity of staining would be increased but without significant binding to normal colon. This should greatly increase the number of tumours detected by radiolabelled antibodies and increase the therapeutic effectiveness of monoclonal antibody drug conjugates.

L.G.D., R.A.R., K.C.B., R.A.M. and R.W.B. are supported by the Cancer Research Campaign, UK. The skilful technical assistance of J. Wright and O. Roberts is gratefully acknowledged.

### References

ABE, K., HAKOMORI, S. & OHABIB, S. (1986). Differential expression of difucosyl Type 2 chain (1e) defined by monoclonal antibody AH6 in different locations of colonic epithelial, various histological types of colonic polyps and adenocarcinomas. *Cancer Res.*, **46**, 2639.

ARMITAGE, N.C., PERKINS, A.C., PIMM, M.V., FARRANDS, P.A., BALDWIN, R.W. & HARDCASTLE, J.D. (1983). The localisation of anti-tumour monoclonal antibody (791T/36) in gastrointestinal tumours. *Br. J. Surg.*, **31**, 407.

BALLANTYNE, K.C., DURRANT, L.G., ARMITAGE, N.C., ROBINS, R.A., BALDWIN, R.W. & HARDCASTLE, J.D. (1986). Binding of a panel of monoclonal antibodies to primary and metastatic colorectal cancer. *Br. J. Cancer*, **54**, 191.

BESTERMAN, J.M. & LOW, R.B. (1983). Endocytosis: a review of mechanisms and plasma membrane dynamics. *Biochem. J.*, **210**, 1.

BRATTAIN, M.G., FINE, W.D., KHATED, F.M., THOMPSON, J. & BRATTAIN, D.E. (1981). Heterogeneity of malignant cells from a human colonic carcinoma. *Cancer Res.*, **41**, 1751.

BROWN, A., FEIZI, T., GOOI, H.C., EMBLETON, M.J., PICARD, J.K. & BALDWIN, R.W. (1983). A monoclonal antibody against human colonic adenoma recognizes difucosylated type-2 blood group chains. *Biosci. Rep.*, **3**, 163.

BROWN, A., ELLIS, I.O., EMBLETON, M.J., BALDWIN, R.W., TURNER, D.B. & HARDCASTLE, J.D. (1984). Immunohistochemical localization of Y hapten and the structurally related H type 2 blood group antigen on large bowel tumours and normal adult tissues. *Int. J. Cancer*, **33**, 727.

BYERS, V.S., PAWLUCZYK, I., BERRY, N. & 5 others (1988). Potentiation of anti-carcinoembryonic antigen immunotoxin cytotoxicity by monoclonal antibodies reacting with co-expressing carcinoembryonic antigen epitopes. *J. Immunol.*, **140**, 4050.

BYERS, V.S., RODVIEN, R., GRANT, K. & 4 others (1989). Phase I study of monoclonal antibody 791T/36-ricin A chain immunotoxin in metastatic colon cancer. *Cancer Res.* (in the press).

CASELLAS, P., BOURRIE, B.J.P., GROS, P. & JANSEN, F.K. (1984). Kinetics of cytotoxicity induced by immunotoxins. *J. Biol. Chem.*, **259**, 9358.

CERIANI, R.L. BLANK, E.W. & PETERSON, J.A. (1987). Experimental immunotherapy of human breast carcinomas implanted in nude mice with a mixture of monoclonal antibodies against human milk fat globule components. *Cancer Res.*, **47**, 532.

DALCHAU, R., KIRLEY, J. & FABRE, S.W. (1980). Monoclonal antibody to a human leucocyte specific membrane glycoprotein probably homologous to the leucocyte common antigen of the rat. *Eur. J. Immunol.*, **10**, 737.

DEXTER, D.L., SPREMULLI, E.N., FLIGIEL, Z. & 4 others (1981). Heterogeneity of cancer cells from a single human colon carcinoma. *Am. J. Med.*, **71**, 949.

DURRANT, L.G., ROBINS, R.A., ARMITAGE, N.C., BROWN, A., BALDWIN, R.W. & HARDCASTLE, J.D. (1986*a*). Association of antigen expression and DNA ploidy in human colorectal tumours. *Cancer Res.*, **46**, 3543.

DURRANT, L.G., ROBINS, R.A., PIMM, M.V. & 4 others (1986*b*). Antigenicity of newly established colorectal cell lines. *Br. J. Cancer*, **53**, 37.

EMBLETON, M.J., GUNN, B., BYERS, V.S. & BALDWIN, R.W. (1981). Antitumour reactions of monoclonal antibody against a human osteogenic sarcoma cell line. *Br. J. Cancer*, **43**, 582.

ERNST, C.S., SHEN, J.-W., LITWIN, S., HERLYN, M., KOPROWSKI, H, & SEARS, H.F. (1986). Multiparameter evaluation of the expression in situ of normal and tumor-associated antigens in human colorectal carcinoma. *J. Natl Cancer Inst.*, **77**, 387.

FARRANDS, P.A., PERKINS, A.C., PIMM, M.V., HARDY, J.G., BALDWIN, R.W. & HARDCASTLE, J.D. (1982). Radioimmunodetection of human colorectal cancers using an anti-tumour monoclonal antibody. *Lancet*, **ii**, 397.

GARNETT, M.C. & BALDWIN, R.W. (1986*a*). An improved synthesis of a methotrexate-albumin-791T/36 monoclonal antibody conjugate cytotoxic to osteogenic sarcoma cell lines. *Cancer Res.*, **46**, 2407.

GARNETT, M.C. & BALDWIN, R.W. (1986*b*). Endocytosis of a monoclonal antibody recognising a cell surface glycoprotein antigen visualised using fluorescent conjugates. *Eur. J. Cell Biol.*, **41**, 214.

HERLYN, M., STEPLEWSKI, Z., HERLYN, D. & KOPROWSKI, H. (1979). Colorectal carcinoma specific antigen: detection by means of monoclonal antibodies. *Proc. Natl Acad. Sci. USA* **76**, 1438.

KRIZAN, Z., MURRAY, S.L., HERSH, E.M. & 4 others (1985). Increased labeling of human melanoma cells in vitro using combinations of monoclonal antibodies recognising separate cell surface antigenic determinants. *Cancer Res.*, **45**, 4904.

LINDHOLM, L., HOLMGREN, J., SVENNERHOLM, L. & 5 others (1983). Monoclonal antibodies against gastrointestinal tumour-associated antigens isolated as monosialo gangliosides. *Int. Arch. Allergy Appl. Immunol.*, **71**, 178.

LLOYD, K.O., LARSON, G., STROMBERG, N., THURIN, J. & KARLSSON, K.A. (1983). Mouse monoclonal antibody F-3 recognises difucosyl type-2 blood group structure. *Immunogenetics*, **17**, 537.

McKENZIE, J.K.L. & FABRE, J.W. (1981). Human Thy-1 unusual localisation and possible functional significance in lymphoid tissues. *J. Immunol.*, **126**, 843.

MAKIN, C.A., BOBROW, L.G. & BODMER, W.F. (1984). Monoclonal antibody to cytokeratin for use in routine histopathology. *J. Clin. Pathol.*, **37**, 975.

PETRICCIANI, J.C., SMITH, P., EARLEY, E.M., LEVENBOOK, I. & NOGUCHI, P. (1982). Characteristics of seven clones of WiDr human colon adenocarcinoma cell line. *In Vitro*, **18**, 492.

PIMM, M.V., PERKINS, A.C., BALLANTYNE, K.C. & 6 others (1986). Experimental and clinical imaging of gastrointestinal carcinomas with $^{111}$In-labelled anti-CEA monoclonal antibodies. *Br. J. Cancer*, **54**, 188.

PRICE, M.R., CAMPBELL, D.G. & BALDWIN, R.W. (1983*a*). Identification of an anti-human osteogenic sarcoma monoclonal-antibody-defined antigen on mitogen-stimulated peripheral blood mononuclear cells. *Scand. J. Immunol.*, **18**, 411.

PRICE, M.R., CAMPBELL, D.G., ROBINS, R.A. & BALDWIN, R.W. (1983*b*). Characteristics of a cell surface antigen defined by an anti-human osteogenic sarcoma monoclonal antibody. *Eur. J. Cancer Clin. Oncol.*, **19**, 81.

PRICE, M.R., EDWARDS, S., JACOBS, E., PAWLUCZYK, I.Z.A., BYERS, V.S. & BALDWIN, R.W. (1987). Mapping of monoclonal antibody-defined epitopes associated with carcinoembyronic antigen, CEA. *Cancer Immunol. Immunother.*, **25**, 10.

SPREMULLI, E.N., SCOTT, C., CAMPBELL, D.E. & 4 others (1983). Characteristics of two metastatic subpopulations originating from a single human colon carcinoma. *Cancer Res.*, **43**, 3828.

STEPLEWSKI, Z & KOPROWSKI, H. (1982). Monoclonal antibody development in the study of colorectal carcinoma-associated antigens. In *Methods of Cancer Research 20*, Busch, H. & Yeoman, L.C. (eds) p. 285. Academic Press: London.


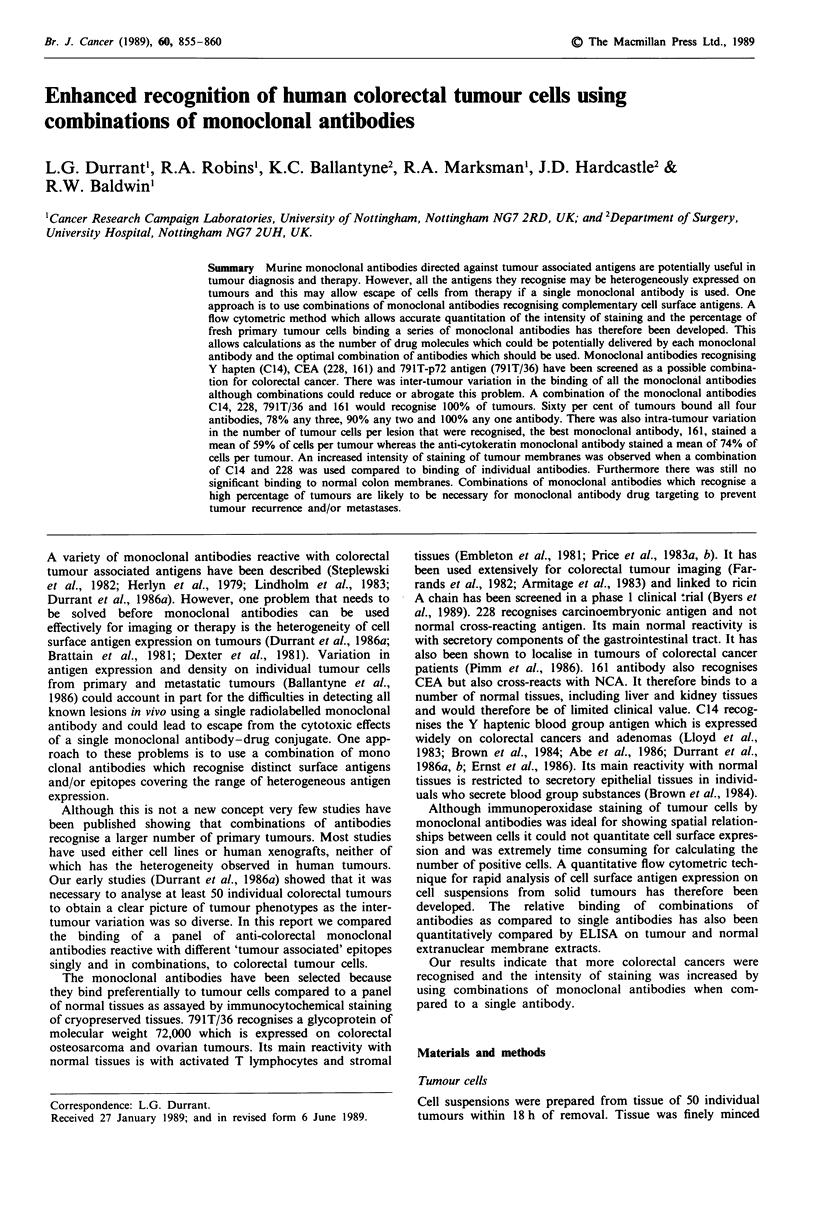

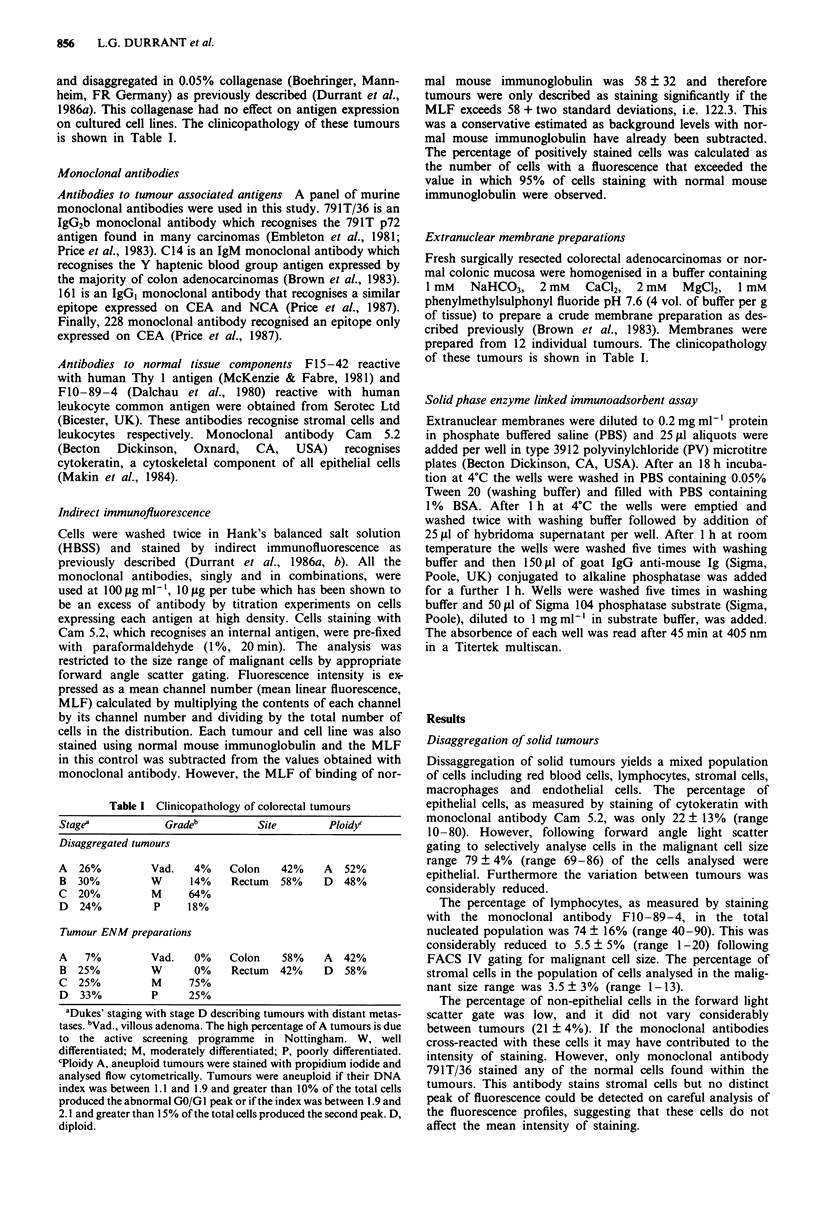

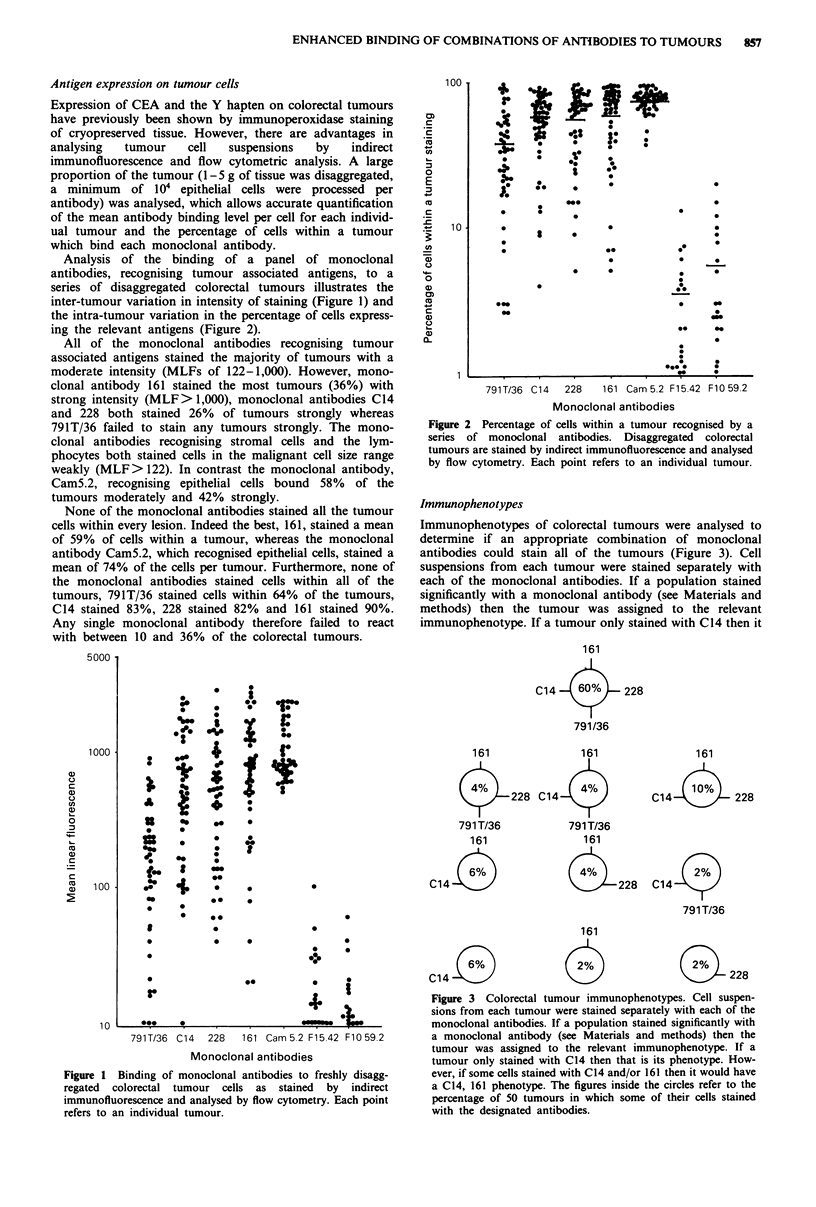

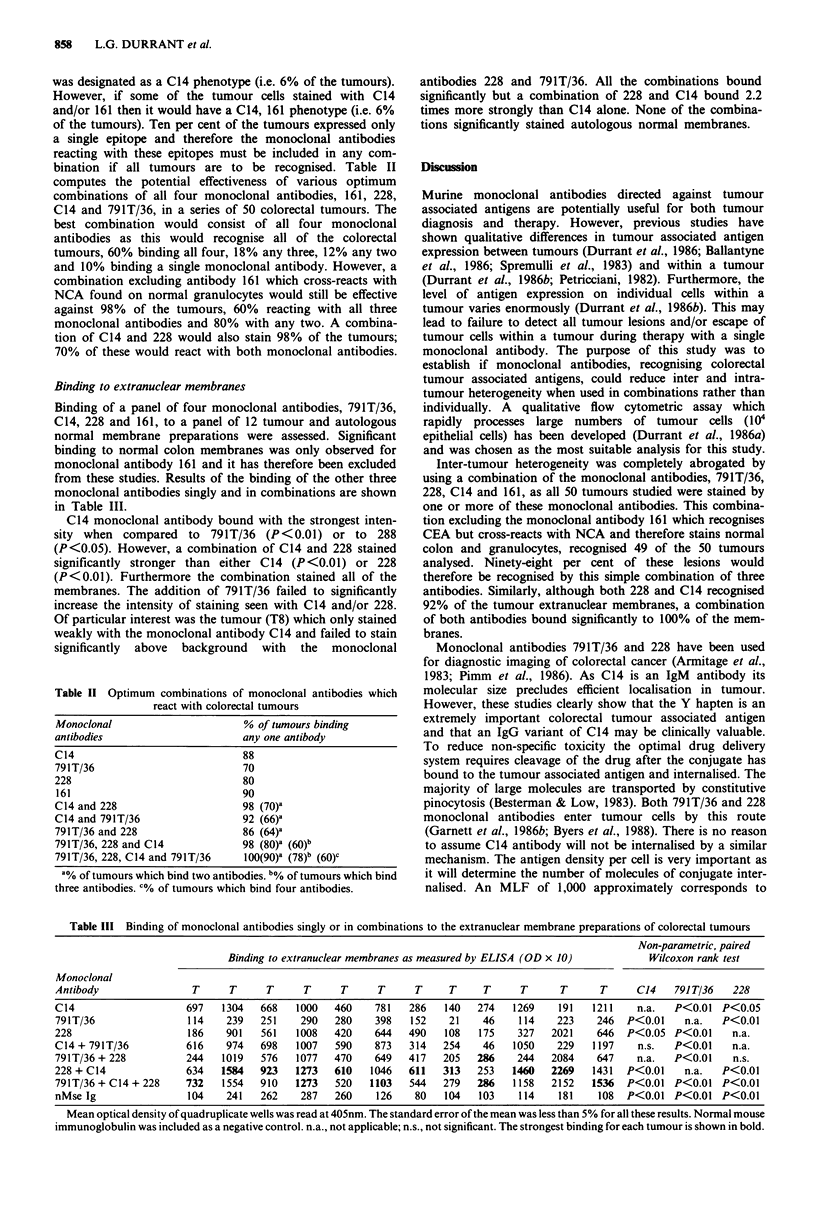

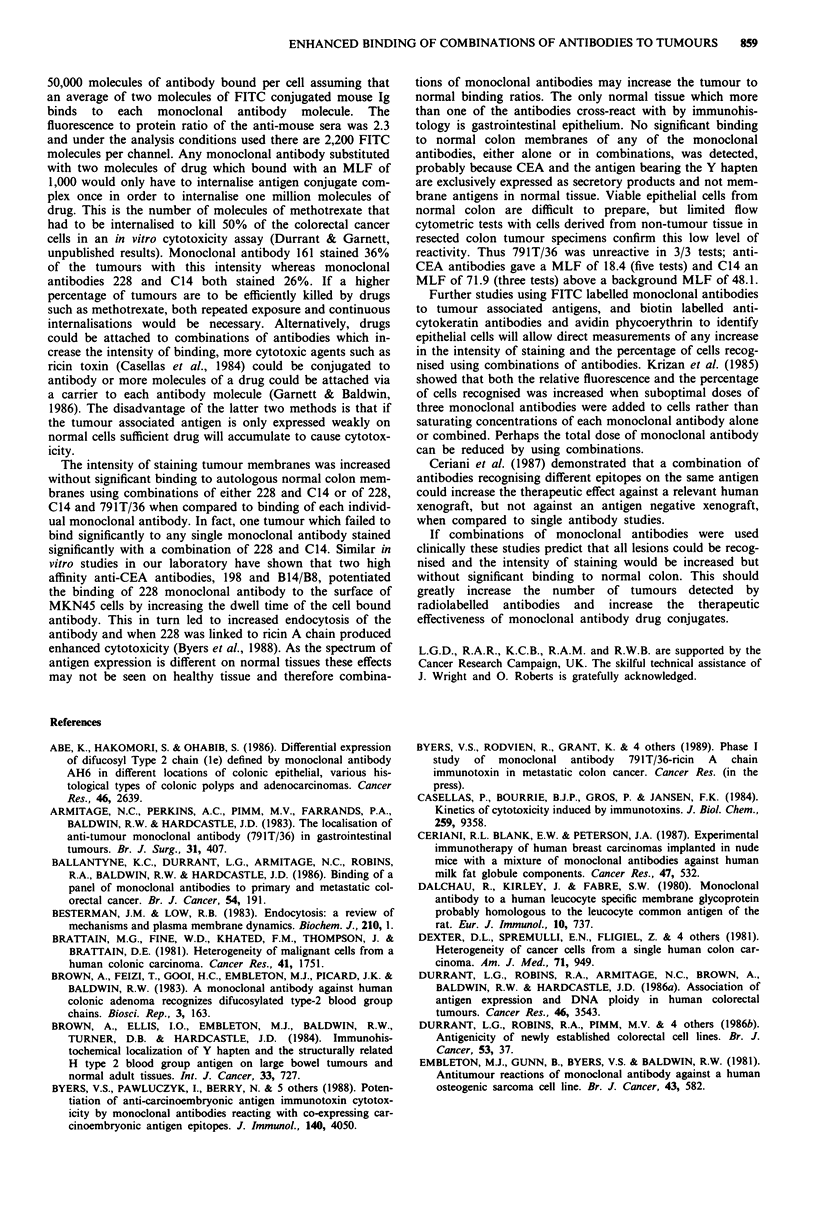

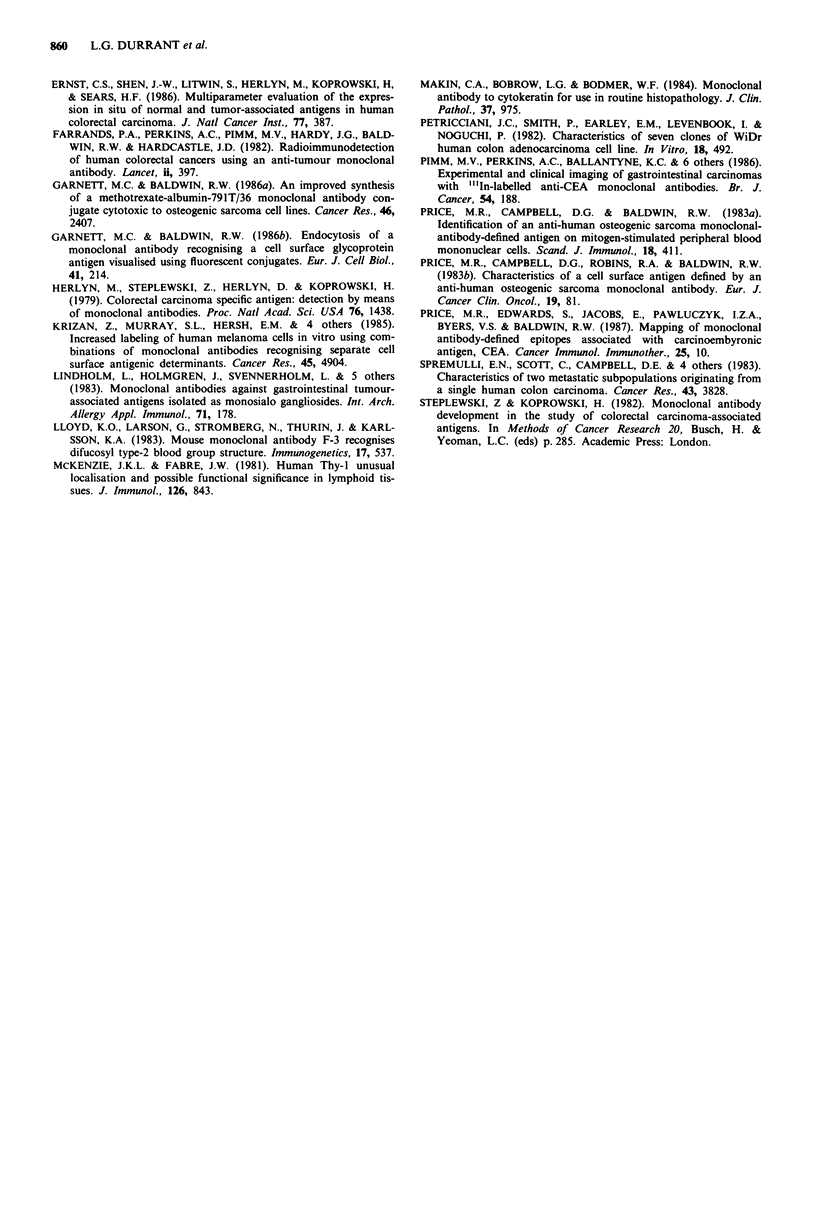


## References

[OCR_00833] Abe K., Hakomori S., Ohshiba S. (1986). Differential expression of difucosyl type 2 chain (LeY) defined by monoclonal antibody AH6 in different locations of colonic epithelia, various histological types of colonic polyps, and adenocarcinomas.. Cancer Res.

[OCR_00852] Besterman J. M., Low R. B. (1983). Endocytosis: a review of mechanisms and plasma membrane dynamics.. Biochem J.

[OCR_00855] Brattain M. G., Fine W. D., Khaled F. M., Thompson J., Brattain D. E. (1981). Heterogeneity of malignant cells from a human colonic carcinoma.. Cancer Res.

[OCR_00866] Brown A., Ellis I. O., Embleton M. J., Baldwin R. W., Turner D. R., Hardcastle J. D. (1984). Immunohistochemical localization of Y hapten and the structurally related H type-2 blood-group antigen on large-bowel tumours and normal adult tissues.. Int J Cancer.

[OCR_00860] Brown A., Feizi T., Gooi H. C., Embleton M. J., Picard J. K., Baldwin R. W. (1983). A monoclonal antibody against human colonic adenoma recognizes difucosylated Type-2-blood-group chains.. Biosci Rep.

[OCR_00873] Byers V. S., Pawluczyk I., Pawlucyzk I., Berry N., Durrant L., Robins R. A., Garnett M. C., Price M. R., Baldwin R. W. (1988). Potentiation of anti-carcinoembryonic antigen immunotoxin cytotoxicity by monoclonal antibodies reacting with co-expressed carcinoembryonic antigen epitopes.. J Immunol.

[OCR_00890] Ceriani R. L., Blank E. W., Peterson J. A. (1987). Experimental immunotherapy of human breast carcinomas implanted in nude mice with a mixture of monoclonal antibodies against human milk fat globule components.. Cancer Res.

[OCR_00896] Dalchau R., Kirkley J., Fabre J. W. (1980). Monoclonal antibody to a human leukocyte-specific membrane glycoprotein probably homologous to the leukocyte-common (L-C) antigen of the rat.. Eur J Immunol.

[OCR_00902] Dexter D. L., Spremulli E. N., Fligiel Z., Barbosa J. A., Vogel R., VanVoorhees A., Calabresi P. (1981). Heterogeneity of cancer cells from a single human colon carcinoma.. Am J Med.

[OCR_00907] Durrant L. G., Robins R. A., Armitage N. C., Brown A., Baldwin R. W., Hardcastle J. D. (1986). Association of antigen expression and DNA ploidy in human colorectal tumors.. Cancer Res.

[OCR_00918] Embleton M. J., Gunn B., Byers V. S., Baldwin R. W. (1981). Antitumour reactions of monoclonal antibody against a human osteogenic-sarcoma cell line.. Br J Cancer.

[OCR_00925] Ernst C. S., Shen J. W., Litwin S., Herlyn M., Koprowski H., Sears H. F. (1986). Multiparameter evaluation of the expression in situ of normal and tumor-associated antigens in human colorectal carcinoma.. J Natl Cancer Inst.

[OCR_00933] Farrands P. A., Perkins A. C., Pimm M. V., Embleton M. J., Hardy J. D., Baldwin R. W., Hardcastle J. D. (1982). Radioimmunodetection of human colorectal cancers by an anti-tumour monoclonal antibody.. Lancet.

[OCR_00937] Garnett M. C., Baldwin R. W. (1986). An improved synthesis of a methotrexate-albumin-791T/36 monoclonal antibody conjugate cytotoxic to human osteogenic sarcoma cell lines.. Cancer Res.

[OCR_00943] Garnett M. C., Baldwin R. W. (1986). Endocytosis of a monoclonal antibody recognising a cell surface glycoprotein antigen visualised using fluorescent conjugates.. Eur J Cell Biol.

[OCR_00949] Herlyn M., Steplewski Z., Herlyn D., Koprowski H. (1979). Colorectal carcinoma-specific antigen: detection by means of monoclonal antibodies.. Proc Natl Acad Sci U S A.

[OCR_00953] Krizan Z., Murray J. L., Hersh E. M., Rosenblum M. G., Glenn H. J., Gschwind C. R., Carlo D. J. (1985). Increased labeling of human melanoma cells in vitro using combinations of monoclonal antibodies recognizing separate cell surface antigenic determinants.. Cancer Res.

[OCR_00959] Lindholm L., Holmgren J., Svennerholm L., Fredman P., Nilsson O., Persson B., Myrvold H., Lagergård T. (1983). Monoclonal antibodies against gastrointestinal tumour-associated antigens isolated as monosialogangliosides.. Int Arch Allergy Appl Immunol.

[OCR_00967] Lloyd K. O., Larson G., Strömberg N., Thurin J., Karlsson K. A. (1983). Mouse monoclonal antibody F-3 recognizes the difucosyl type-2 blood group structure.. Immunogenetics.

[OCR_00974] Makin C. A., Bobrow L. G., Bodmer W. F. (1984). Monoclonal antibody to cytokeratin for use in routine histopathology.. J Clin Pathol.

[OCR_00969] McKenzie J. L., Fabre J. W. (1981). Human thy-1: unusual localization and possible functional significance in lymphoid tissues.. J Immunol.

[OCR_00979] Petricciani J. C., Smith P., Earley E. M., Levenbook I., Noguchi P. (1982). Characteristics of seven clones of the WiDr human colon adenocarcinoma cell line.. In Vitro.

[OCR_00990] Price M. R., Campbell D. G., Baldwin R. W. (1983). Identification of an anti-human osteogenic sarcoma monoclonal-antibody-defined antigen on mitogen-stimulated peripheral blood mononuclear cells.. Scand J Immunol.

[OCR_00996] Price M. R., Campbell D. G., Robins R. A., Baldwin R. W. (1983). Characteristics of a cell surface antigen defined by an anti-human osteogenic sarcoma monoclonal antibody.. Eur J Cancer Clin Oncol.

[OCR_01002] Price M. R., Edwards S., Jacobs E., Pawluczyk I. Z., Byers V. S., Baldwin R. W. (1987). Mapping of monoclonal antibody-defined epitopes associated with carcinoembryonic antigen, CEA.. Cancer Immunol Immunother.

[OCR_01008] Spremulli E. N., Scott C., Campbell D. E., Libbey N. P., Shochat D., Gold D. V., Dexter D. L. (1983). Characterization of two metastatic subpopulations originating from a single human colon carcinoma.. Cancer Res.

